# Reduced Pleasant Touch Appraisal in the Presence of a Disgusting Odor

**DOI:** 10.1371/journal.pone.0092975

**Published:** 2014-03-24

**Authors:** Ilona Croy, Silvia D' Angelo, Håkan Olausson

**Affiliations:** Department of Clinical Neurophysiology, Sahlgrenska University Hospital, University of Gothenburg, Gothenburg, Sweden; Duke University, United States of America

## Abstract

**Objectives:**

Odors are powerful emotional stimuli influencing mood, attention and behavior. Here we examined if odors change the perception of pleasant touch. In line with the warning function of the olfactory system, we proposed that especially unpleasant odors will reduce touch pleasantness, presumably through a disgust-related mechanism.

**Methods:**

Forty-five healthy participants (mean age 23.3 +/− 3years SD, 24 females) were presented to slow (3 cm/s) and fast (30 cm/s) brush stroking delivered by a robot to the forearm. Touch pleasantness under the influence of an unpleasant odor (Civette, smelling like feces) and an intensity matched pleasant odor (Rose) was compared to an odorless control condition. In a pilot study with 30 participants (mean age 25.9 +/−6 years, 21 females), the odors were matched according to their intensity, and we studied the influence of disgust sensitivity on the perception of 4 different odor qualities.

**Results:**

The unpleasant odor decreased touch pleasantness for both stroking velocities compared to the odorless control (p<0.005) whereas the rose odor did not change touch pleasantness significantly. Disgust sensitivity was correlated with the modulation of touch pleasantness. The pilot study revealed a significant correlation between disgust sensitivity and the perception of the unpleasant odor qualities (r = −0.56; p = 0.007), but not with any of the other odors.

**Conclusion:**

Unpleasant odors are powerful in modulating touch pleasantness, and disgust might be a moderating variable.

## Introduction

We are surrounded by thousands of odors that are processed with every breath we take and add to the comprehensive impression of the environment. Consequently, odors have the potential to influence mood [1], [2], attention [3] and behavior [4], [5]. Moreover, odors influence the perception of other sensory stimuli. Most obviously, odors interact with taste stimuli in forming a specific flavor of food [6], but odors also change the perception of sounds [7], pictures [5] and discriminative touch [8], [9]. Odorized fabrics feel rougher or softer, depending on the odor [8] and odors of shampoos influence the perceived texture of the hair [9]. We aimed to analyze whether odors also have the potential to moderate the perception of the affective aspect of touch.

The sensation of pleasant touch, as it happens during stroking and caressing, is critical for social development [10] and is presumably coded by specialized unmyelinated C fibers (C-tactile, CT), that are found in the hairy skin [11], [12]. In experimental conditions, pleasant touch is typically given by a brush moving slowly over the hairy skin of the forearm. Using this paradigm, it was found that the relation between stroking speed and firing rate of single unit CTs follows an inversed u-shaped curve with an optimal velocity of 1–10 cm/s. Further, the CT firing rate is highly correlated to touch pleasantness ratings [13] while myelinated Aβ afferents increase their firing rate monotonically with stroking speed [13]. Cross modal interactions between CT targeted touch and other sensory input have been recently studied using facial expressions [14]. Here, participants rated CT targeted touch significantly more pleasant when presented with a happy compared to an angry face. More studies deal with cross modal interactions on the perception of texture. The perception of textures can be influenced by odors [8], [9], tones [15] and visual stimuli [16] and such cross modal interactions even change processing in early somatosensory cortices [16].

The perception of pleasantness is an emotional appraisal and odors are very powerful emotional stimuli. It is therefore plausible to assume that the sensory integration of an odor with pleasant touch changes touch perception. Interestingly, not every basic emotion can be evoked easily by odors, but disgust and happiness are reliably evoked by the sense of smell [17]–[19]. Specific odors are perceived differently based on the individual's experience and cultural background. However, some classes of odors seem to evoke similar emotions across a wide range of persons: The odor of food and flowers evoking happiness and the odor of feces evoking disgust [18], [20], and it has been suggested that the olfactory system warns about microbial danger by evoking disgust [21]. The behavioral consequence would be not to touch or eat such an object. In fact, people avoid touching objects that they perceive disgusting [22] and we assume, that people also dislike being touched, if the touch is associated with a disgusting odor.

Feces are believed to be cross-culturally perceived as disgusting [23]. Therefore a feces-like odor was used for the modulation of pleasant touch and compared to a pleasant odor and an odorless control. The perceived unpleasantness of a feces odor varies among persons [24]. As we assume that feces evoke disgust, the general disgust sensitivity of the participants may explain some of this variation. Disgust sensitivity was measured with the use of questionnaires [25], [26].

Two studies were carried out. Study one was a pilot study performed in order to match the intensity of odors and additionally to examine the influence of general disgust sensitivity on odor valence perception. Study two analyzed the impact of a pleasant and an unpleasant odor on the perception of two types of pleasant touch stimuli; slow brush stroking targeted to activate CT fibers and fast brush stroking targeted to activate Aβ fibers. We expected a decrease of touch pleasantness in the presence of a feces odor. As CT-targeted touch is hypothesized to code social relevant touch [11], [12], we expected a stronger impact of the feces odor here compared to the Aβ-targeted touch.

## Methods

### Ethics Statement

The investigations were performed according to the Declaration of Helsinki on Biomedical Research Involving Human Subjects. The protocol was approved by the central ethics committee in Gothenburg, Sweden. Written informed consent was obtained following explanation of the study.

### Study 1

#### Sample

Thirty volunteers (21 female, 9 male, age range 19 to 40 years, mean 25.9 +/−6.0 years SD) participated. Most of them were undergraduate psychology students.

#### Questionnaires

Before the experiment started, all participants filled out a questionnaire about disgust sensitivity. The Disgust Scale consists of 32 items assessing the individual sensitivity to disgusting stimuli [25], [26].

#### Procedure

After filling out the questionnaire, all participants received six different qualities of odors (all provided by Firmenich (Kerpen, Germany): Coconut, Rose, Flower, Vanilla, Aloe and Civette (smelling like feces). All odors were diluted in 1,2-propanediol in 3 to 4 different concentrations (compare table 1). Rose and Civette were presented at four concentrations, in order to be able to select matching intensities for study 2 from a wider range. To avoid visual distraction, the odor dilutions were kept in brown flasks (50 ml, diameter of opening 2.5 cm) and each odor was absorbed on a piece of cotton to ensure a better exchange with the air. Each flask was presented for about 2sec under the participant's nostrils in a randomized order. The participants were instructed to smell each odor and then evaluate their intensity and pleasantness, using an 11 point scale (pleasantness: -5 (extremely unpleasant) to 5 (extremely pleasant); intensity: 0 (not intense at all) to 10 (extremely intense).

**Table 1:** Ratings of odor pleasantness and intensity and correlation with disgust sensitivity.

		Odor ratings				Correlation between disgust sensitivity and	
Odor	Concentration	Odor pleasantness		Odor intensity		Odor pleasantness	Odor Intensity
		Mean	SD	Mean	SD		
Aloe	low (0.5%)	2.8	1.3	4.0	2.2		
	middle (1.0%)	2.3	1.2	5.6	2.1		
	high (1.8%)	2.1	1.9	4.9	2.1		
	***combined***	2.4	1.2	4.8	1.7	.37	−0.24
Civette	**low (0.7%)**	−2.5	2.0	6.1	2.6		
	middle (2.2%)	−2.8	2.5	7.0	2.3		
	high (6.6%)	−3.7	1.6	7.8	2.2		
	very high (20%)	−3.5	1.4	7.4	2.1		
	***combined***	−3.0	1.4	7.0	2.1	−.56*	.44*
Coconut	low (1.8%)	2.4	1.2	5.3	2.2		
	middle (5.5%)	2.2	1.7	6.7	1.6		
	high (16.6%)	1.8	2.1	6.3	1.9		
	***combined***	2.1	1.3	6.1	1.5	−0.14	−0.20
Flower	low (1.8%)	2.3	1.7	4.7	2.5		
	middle (5.5%)	1.9	1.8	5.7	2.7		
	high (16.6%)	1.5	1.7	6.4	1.8		
	***combined***	1.9	1.5	5.6	1.9	−0.23	−0.03
Rose	low (1.8%)	2.0	1.4	5.8	2.1		
	middle (5.5%)	2.2	1.7	6.0	1.8		
	**high (18.5%)**	1.5	1.8	6.3	2.0		
	very high (50%)	1.7	1.7	6.6	1.9		
	***combined***	1.9	1.4	6.0	1.6	0.02	−0.09
Vanilla	low (0.5%)	0.1	2.5	5.8	2.4		
	middle (1.0%)	0.4	2.4	5.9	1.7		
	high (1.8%)	0.1	2.6	6.2	1.8		
	***combined***	0.2	2.3	6.0	1.7	−0.19	−0.02

*Note: Combined odor ratings encompass all three odor ratings or for Civette all except the 20% concentration and for Rose all except the 1.8% concentration. Civette (0.7%, printed in Bold) and Rose (18.5% printed in Bold) were selected for study II.* * … p_bonf_ <0.05.

#### Statistical analysis

Statistical analyses were performed using SPSS version 21 (*IBM*, Chicago, USA). The intensity ratings of Civette were compared with the intensity ratings of Rose at different concentrations with the help of t-test for paired samples. In order to correlate the questionnaire scores with the odor ratings, we combined odor rating scores for each odor quality. Therefore all three concentrations of each odor quality were averaged. For Civette, however, which was presented at four concentrations, the highest concentration was not taken into account for the average. For Rose, the lowest concentration was left out. This was done, in order to obtain combinations with similar intensity ratings. An ANOVA for repeated measurements (all 6 odor qualities) was carried out to examine intensity differences in the combined odor ratings. The 6 combined odor ratings were correlated with the results from the disgust sensitivity questionnaire using Pearson's coefficient and Bonferroni correction with a factor of 6. Level of significance was set at p = 0.05.

### Study 2

#### Sample

Forty-five volunteers (24 women, 21 men, age range 19 to 32 years, mean age 23.3 +/− 3years) were investigated. Most of the participants were students. They were recruited by public announcements and 14 of them participated in study 1. Normal olfactory function among the participants was ascertained using the “Sniffin’ Sticks” identification test [27] (mean 13.1, +/−1.5). Depressive symptoms were controlled using the BDI questionnaire [28] (scores ranged from 0 to 20, mean 4.2 +/−5.0).

#### Questionnaires

Prior to the experiment, participants filled out the Disgust Scale [25], [26] as well as additional questionnaires about the importance of touch (Tactype) [29] and olfaction [30]. The Tactype consists of 15 items that assesses the attitude towards touching other persons and being touched. The Importance of Olfaction questionnaire measures the daily life use and importance of the sense of smell with 18 items.

#### Procedure

The participants were seated in a comfortable chair in front of a computer screen with their left arm in prone position on a pillow positioned on the left side of the chair. The touch stimuli were applied to the subject's left dorsal forearm by a custom-built robotic device (rotary tactile stimulator, RTS; Dancer Design, UK, stroking 7.5 cm with a 50 mm wide flat, soft watercolor brush made of fine, smooth, goat's hair) driven by LabVIEW (National Instruments, TX). Two different touch stimuli were used: CT targeted brush stroking with a velocity of 3 cm/s and a vertical force of 0.4N and a Aβ targeted brush stroking with a velocity of 30 cm/s and a vertical force of 0.4N [31].

Immediately before each brush stroke, the participants were presented to one of three odor stimuli (Rose, Civette, odorless Control). The odors were presented by a female experimenter for about 2 seconds under the nose of the participants. Based on study I, odors with different valence and similar intensity were selected for presentation: Rose diluted to 18.5% and Civette diluted to 0.7% in 1,2-propanediol. The odors were presented in brown glass flasks, like in study 1. In a third similar flask, the odorless dilution was presented, which served as control stimulus.

Each participant received 18 brush stroking stimuli in three blocks of six stimuli. Each block was under the influence of an odor condition (Rose, Civette, Control) and within each block three CT-targeted and three Aβ targeted brush stroking stimuli were presented. Order of blocks as well as the order of stimuli within a block was randomized across the participants. After each presentation, pleasantness (−5 to 5; extremely unpleasant to extremely pleasant) and intensity (0 to 10, not intense at all to extreme intense) of the touch stimuli was rated on a VAS scale presented on a computer screen.

Before the actual experiment started, the participants were asked to rate the pleasantness and intensity of the odors. Then the touch rating was practiced with the odorless control substance, in order to assure that the participants were rating the touch and not the odor.

#### Statistical analysis

Statistical analyses were performed using SPSS version 21 (*IBM*, Chicago, USA). Pleasantness and intensity of the odors were compared with t-tests for paired measurements. ANOVA for repeated measurements was used to analyze the main effects of odor (3) and velocity (2) and interaction effects on touch pleasantness and intensity ratings. Post hoc testing was performed with 4 paired t-tests and Bonferroni corrected with a factor of 4. The effects of age and sex were analyzed by adding age as a covariate or sex as a between subject factor into the analysis. The individual odor modulation was calculated by subtracting touch ratings under the influence of the odorless control from touch ratings under the influence of the Civette or Rose odor, respectively. This modulation was correlated with disgust sensitivity using Pearson's coefficient. Level of significance was set at p = 0.05.

## Results

### Study 1

The pleasantness and intensity ratings of the 20 odors are displayed in table 1. Rose odor at a concentration of 18.5% and Civette at a concentration of 0.7% were rated as similarly intense (p = 0.75); therefore they were selected for study 2.

**Figure 1:**
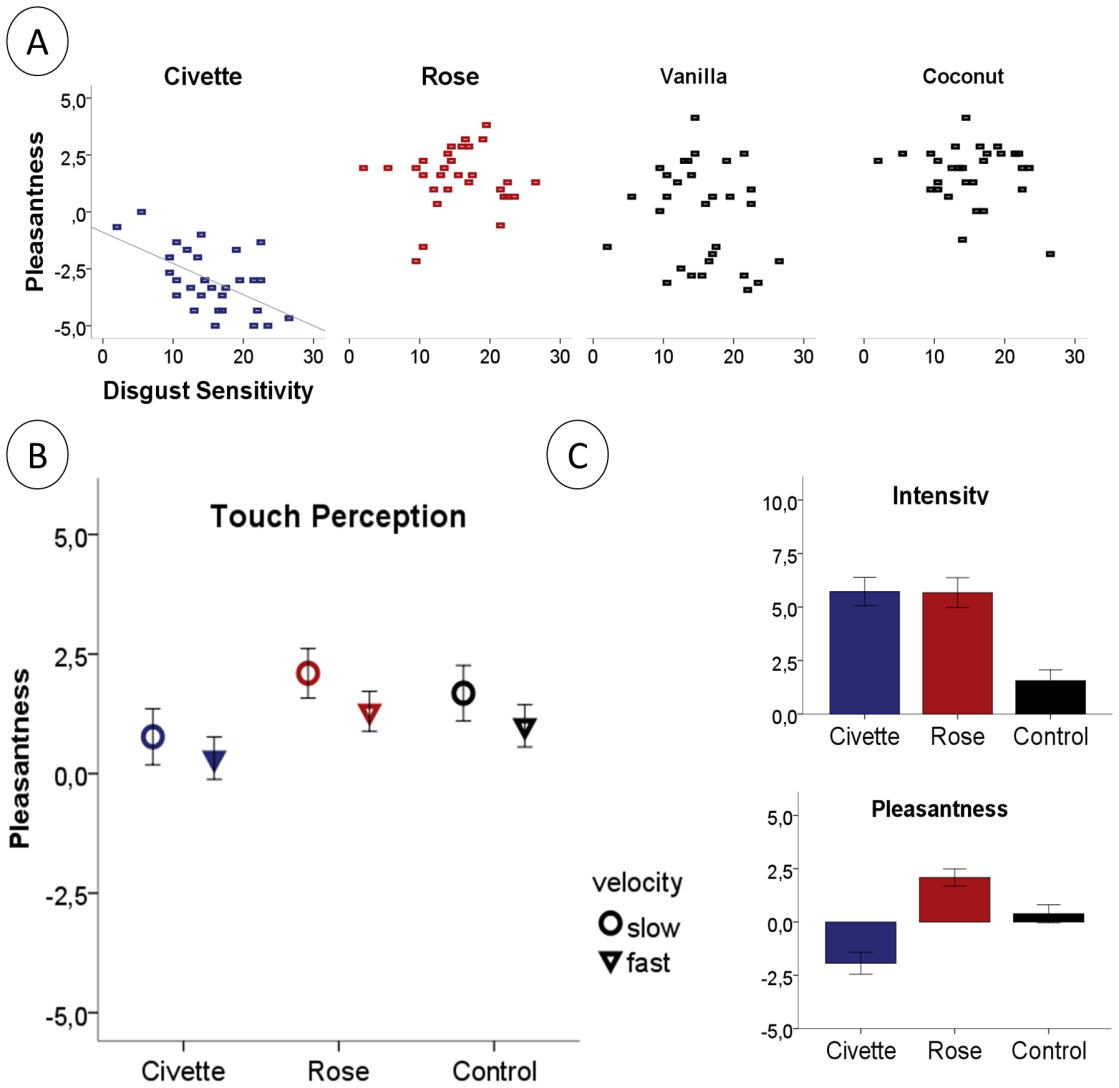
Odor and touch pleasantness. A) Averaged odor pleasantness ratings for 4 odor qualities with similar intensity are displayed in relation to individual disgust sensitivity. There was a significant correlation between the pleasantness of Civette and disgust sensitivity. B) The pleasantness of slow and fast stroking under the influence of Civette, Rose or an odorless Control. Civette significantly decreased the touch pleasantness. C) Rose and Civette did not differ in intensity, but pleasantness.

The 6 combined odor ratings differed in intensity (F[5], [29] = 8.8, p<0.001) with Aloe and Flower being less intense than the other odors. Civette, Rose, Coconut and Vanilla did not differ in perceived intensity, but in pleasantness (F[3], [27] = 81.5, p<0.001, compare figure 1A). There was a significant correlation between disgust sensitivity and the pleasantness of Civette (r = −0.56; p_bonf_ = 0.007, compare figure 1). However, there were no significant correlations between disgust sensitivity and the pleasantness ratings of any of the other odors (r = −0.19 to r = 0.37). For intensity ratings, there was a tendency between disgust sensitivity and the perception of Civette (r = 0.44, p_bonf_ = 0.09), but not for any of the other odors (r = −0.24 to r = +0.03).

### Study II

Confirming the results from study 1, Civette (0.7%) and Rose (18.5%) did not differ in perceived intensity (p = 0.9), but in pleasantness (compare figure 1 and table 2). Rose was perceived as significantly more pleasant than the odorless control (p<0.001) and Civette as significantly more unpleasant compared to the control (p<0.001).

**Table 2:** Odor ratings and touch ratings under the influence of odors.

		odor rating		touch rating under the influence of odors			
				slow (3 cm/s)		fast (30 cm/s)	
		Mean	SD	Mean	SD	Mean	SD
Civette	Pleasantness	−1.9	1.7	0.8	1.9	0.3	1.5
	Intensity	5.7	2.2	4.3	1.9	4.2	1.8
Rose	Pleasantness	2.1	1.3	2.1	1.7	1.3	1.4
	Intensity	5.7	2.3	4.1	2.1	3.8	1.9
Control	Pleasantness	0.4	1.4	1.7	1.9	1.0	1.5
	Intensity	1.6	1.7	4.3	2.2	3.6	1.7

The presentation of odors together with touch modulated the touch perception. There was a significant main effect of odor (F[2], [43] = 24.5, p<0.001) and a significant main effect of stroking velocity (F[1], [44] = 16.1,p<0.001) on ***touch pleasantness***, showing that slow stroking (CT-targeted) was rated more pleasant than the fast (Aβ-targeted) stroking (compare figure 1 and table 2). The interaction between odor and velocity was not significant (F[2,88] = 1.4,p = 0.33). Post hoc testing revealed that Civette decreased the pleasantness of stroking compared to the odorless control (slow: p_bonf_<0.005, fast: p_bonf_<0.005). The Rose odor did not significantly change the perceived pleasantness of stroking compared to the odorless control (slow: p_bonf_ = 0.12, fast: p_bonf_ = 0.15). For ***touch intensity*** perception, there was no significant main effect of odor (F[2,88] = 1.7,p = 0.19) or velocity (F[1], [44] = 2.8,p = 0.1), and no significant interaction effects between odor and velocity (F[2,88] = 2.7,p = 0.08).

**Figure 2:**
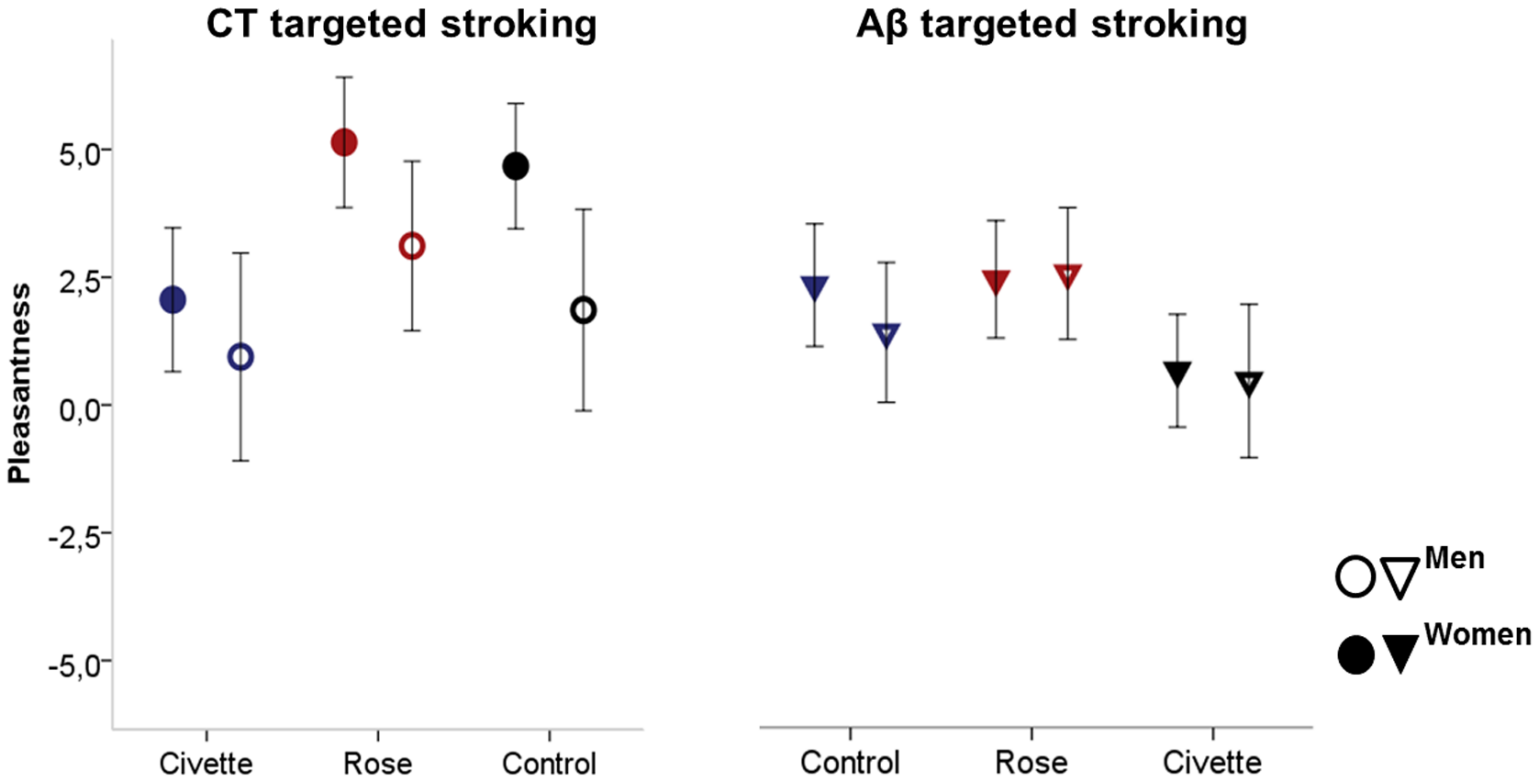
Sex differences in pleasant touch perception. Touch pleasantness of CT targeted slow stroking (left) and Aβ-targeted fast stroking (right) is compared between men (bordered circels and triagels) and women (filled circels and triangels). Women rated the CT targeted touch significantly more pleasant than men.

An additional analysis was carried out examining the influence of gender and age. There were no significant main or interaction effects of age on touch pleasantness (main effect: F1,43 = 0.6, p = 0.8 interaction with odor F2,42 = 1.5, p = 0.1, interaction with velocity F1,43 = 0.5, p = 0.8) or intensity (main effect: F1,43 = 2.8, p = 0.1, interaction with odor F2,42 = 1.9, p = 0.2, interaction with velocity F1,43 = 1.1, p = 0.3) and no significant effects of sex (*intensity*: main effect: F1,43 = 0.04, p = 0.8, interaction with odor F2,42 = 0.4, p = 0.6, interaction with velocity F1,43 = 0.1, p = 0.7; *pleasantness*: main effect: F1,43 = 2.0, p = 0.2, interaction with odor F2,42 = 1.7, p = 0.2), except for a sex*velocity interaction for touch pleasantness (F1,43 = 7.6, p = 0.007, compare figure 2). Women rated the slow touch more pleasant than men (p = 0.04), irrespective of the odor-condition. For the fast velocity, no such effect was observed.

Eighty percent of the participants rated slow touch less pleasant under the influence of Civette than under the influence of the odorless control. Sixty-seven percent of the participants rated slow touch more pleasant under the influence of rose than under the influence of the odorless control. There was a significant correlation between disgust sensitivity and the reduction of touch pleasantness for both odors (Civette r = −.30, p = .04, Rose r = −.32, p = .03), implying that participants who had higher disgust sensitivity were more negatively influenced in their touch ratings by the odors. There were no significant correlations with the questionnaires about the importance of touch or olfaction.

## Discussion

In line with our hypothesis, pleasant touch perception was modulated by olfactory stimuli. Thereby affective touch can be added to the list of gustatory, auditory and visual stimuli [5], [7], [32] that interact with odors. For discriminative tactile stimuli (such as fabrics or hair), it had been shown before, that active touch perception is influenced by odors [8], [9].

Unpleasant odors are usually processed faster and evoke larger P2 amplitudes in event related potentials than pleasant ones, indicating a higher significance [33]. In line with this, Rose had a lower impact for modulating touch pleasantness than Civette. The feces-like smelling Civette odor reduced touch pleasantness, while the pleasant rose odor did not enhance it significantly.

Comparing the two stroking velocities, we found significantly higher pleasantness ratings for the slow, CT-targeted stroking stimulus, confirming previous results [31], [34]. Furthermore, we found sex differences with women rating the CT-targeted, but not the Aβ-targeted, touch significantly more pleasant than men, irrespective of the preceding odor. Civette had a slightly stronger impact on the CT-targeted compared to the Aβ-targeted touch. However, the effects were very small and not significant. Previous studies show, that odors have the potential to modulate Aβ targeted touch [8], [9]. However, from our results, we cannot say if the modulation is similar or different for the two types of touch. For both stroking qualities, Civette decreased touch pleasantness and Rose did not modulate it significantly.

This differential impact of the odor qualities could be due to a methodological problem. The perception of pleasant touch was already predictably high in the control condition. This ceiling effect makes modulations to the bottom more likely than to the top. In to order estimate the influence of ceiling effects, the analysis was repeated under exclusion of participants with low space for modulation (touch pleasantness ratings higher than 2.5 or lower than −2.5 in the control condition). The analysis with the remaining 27 participants who were less prone to ceiling effects, confirmed the results: Civette reduced touch pleasantness significantly (p<0.001) and Rose did not enhance it (p = 0.18) compared to the control. We assume that despite being a potential influencing factor, any ceiling effect is not the main drive for the different effect of Civette and Rose. Interestingly, it has been shown, in a design similar to ours, that unpleasant odors influence attractiveness perception [35]. In this study, women rate the attractiveness of male faces significantly lower, if they simultaneously smell unpleasant rated odors (rubber odor and body odor). Pleasant rated odors (Geranium and Male fragrance), on the other hand, had no significant impact on the facial attractiveness.

It is no new idea that unpleasant stimuli have a higher potential for modulating perception in general. We have reasons to assume, that disgust is a moderator for such interactions in the domain of olfaction. Disgust has been described as a disease avoiding mechanism [36] and is easily triggered by odors [2], [21]. Consequently, certain odors are perceived disgusting across various cultures [37] and it has been shown before, that the feces like Civette odor evokes disgust [24]. Odor and touch presumably interact in the anterior insular cortex, which is reliably activated in olfactory [38] and pleasant touch stimuli [39] and considered important for the integration of multimodal sensory input [40]. The anterior insular cortex is also highly activated in the presence of disgust and has been suggested to integrate disgust response with olfaction [41].

An alternative explanation of our results refers to attention. In the odor conditions, attention had to be shared between two sensory inputs: tactile and olfactory. It is possible, that the unpleasant Civette odor biased attention more than the pleasant Rose odor since subjects respond faster to congruent than incongruent odor-touch interactions [42]. However, we do not think that attention differences fully explain our results. First, attention was required in both odor conditions, but for the Rose odor, touch pleasantness was not decreased. Second, intensity ratings are also dependent on attention [43] but we found no significant differences between the touch conditions.

Applied to daily life, our data indicates, that humans want to be stroked less, if the stroker has an awful smell. That is not surprising. Yet, we were able to show this effect under controlled laboratory conditions. It is noteworthy, that odors and tactile stimuli were delivered in temporal proximity, but from different sources (RTS robot vs bottles containing the odors). Under real life conditions, the stroking and the odor would typically come from the same source with the possibility of higher crossmodal effects.

Individual disgust ratings towards Civette were not examined, which limits the interpretation of our data. However, a more general measure of disgust sensitivity was obtained with the use of a questionnaire. People vary in how prone they are in feeling disgust and the individual disgust sensitivity correlates with activation of the anterior insula evoked by disgusting stimuli [44]. In line with this, we found in study I that disgust sensitivity was related to the valence of the feces-like odor, but not to the valence of any of the other more pleasant odors. Study two showed, that touch appraisal per se was unrelated to disgust sensitivity. Yet, the modulation of pleasant touch perception by odors was correlated with disgust sensitivity. Interestingly, this was the case for both odors: The higher the disgust sensitivity, the more disturbed was touch appraisal by odors. It is possible that persons with high disgust sensitivity are in general more sensitive to potential disturbances of pleasant touch perception.

We conclude that touch perception is modulated by odors. An unpleasant odor has the potential to reduce the perceived pleasantness of touch and disgust sensitivity may facilitate the interaction of pleasant touch perception and olfaction.
